# Detection of Coronaviruses Using RNA Toehold Switch Sensors

**DOI:** 10.3390/ijms22041772

**Published:** 2021-02-10

**Authors:** Soan Park, Jeong Wook Lee

**Affiliations:** 1 Department of Chemical Engineering, Pohang University of Science and Technology, 77 Cheongam-ro, Nam-gu, Pohang, Gyeongbuk 37673, Korea; soan114@postech.ac.kr; 2 School of Interdisciplinary Bioscience and Bioengineering, Pohang University of Science, 77 Cheongam-ro, Nam-gu, Pohang, Gyeongbuk 37673, Korea

**Keywords:** middle east respiratory syndrome coronavirus (MERS-CoV), severe acute respiratory syndrome coronavirus 2 (SARS-CoV-2), molecular diagnostics, reverse transcription loop-mediated isothermal amplification (RT-LAMP)

## Abstract

A rapid, sensitive and simple point-of-care (POC) nucleic acid diagnostic test is needed to prevent spread of infectious diseases. Paper-based toehold reaction, a recently emerged colorimetric POC nucleic acid diagnostic test, has been widely used for pathogen detection and microbiome profiling. Here, we introduce an amplification method called reverse transcription loop-mediated amplification (RT-LAMP) prior to the toehold reaction and modify it to enable more sensitive and faster colorimetric detection of RNA viruses. We show that incorporating the modified RT-LAMP to the toehold reaction detects as few as 120 copies of coronavirus RNA in 70 min. Cross-reactivity test against other coronaviruses indicates this toehold reaction with the modified RT-LAMP is highly specific to the target RNA. Overall, the paper-based toehold switch sensors with the modified RT-LAMP allow fast, sensitive, specific and colorimetric coronavirus detection.

## 1. Introduction

Among four genera of coronaviruses, humans can be only infected with the members of alpha and beta coronaviruses. Many of these human coronaviruses such as HCoV-229E, HCoV-OC43, HCoV-NL63 and HCoV-HKU1, leading to common colds, are not regarded as severe pathogens [1]. However, in recent years, three beta coronavirus variants, including severe acute respiratory syndrome coronavirus (SARS-CoV), middle east respiratory syndrome coronavirus (MERS-CoV) and severe acute respiratory syndrome coronavirus 2 (SARS-CoV-2) caused more severe and even fatal infectious diseases with high morbidity and mortality. Currently, the world faces unprecedented challenges by COVID-19 caused by SARS-CoV-2 and this fast-spreading pandemic disease is threatening global health. To prevent the spread of such viruses, a rapid and simple point-of-care (POC) diagnosis is needed [2,3].

Real-time reverse transcription polymerase chain reaction (rRT-PCR) is the most widely used laboratory method for RNA virus detection owing to its sensitivity and selectivity [1,4]. However, rRT-PCR requires a series of specialized equipment and diagnostic expertise to perform the diagnostic reaction, so that it cannot be used as a POC diagnosis in the field. A serological test can be an optimal solution to such on-site diagnostics. However, antibody concentrations in the body are typically low to detect in the early stage of RNA virus infection [5]. Even if the antibody concentration is increased, cross-reactivity of antibodies caused by other coronavirus infections often reduces the selectivity of serological tests [6,7].

To overcome the limitation, nucleic acid-based on-site detection methods that combine isothermal nucleic acid amplification have been suggested recently [8,9,10]. One of the pioneering technologies is a paper-based nucleic acid diagnostic method, enabling portable and colorimetric detection of RNA viruses [11]. The sequence-specific detection mechanism relies on a synthetic riboswitch called a toehold switch. In the toehold switch, the translational initiation site of a reporter protein is sequestered in the stem-loop RNA secondary structure. The stem-loop structure can only be unfolded when it meets a trans-trigger sequence that complements a part of the toehold switch. By configuring toehold and trigger pairs according to a target nucleic acid sequence, the system can be used to detect the presence of the target sequence in a sample, simply monitoring the reporter expression [12].

In order to enhance the system’s sensitivity, nucleic acid sequence-based amplification (NASBA) method as an isothermal amplification method has been coupled to the toehold reaction [12]. However, NASBA takes 1–3 h for amplification and the detection limit of NASBA is relatively low femtomolar concentration [12], so an alternative isothermal amplification method is needed to improve the portable and colorimetric sequence-specific detection system.

In this study, we introduced reverse transcription loop-mediated amplification (RT-LAMP) for RNA target to the toehold reaction as an alternative to NASBA. To obtain RNA as an amplification product after the LAMP DNA amplification, we strategically modified a LAMP primer to include a T7 promoter upstream of the amplicon sequence. This modified LAMP amplification strategy was coupled with the paper-based toehold reaction. To test the system’s usability and adaptability, we applied this method to detect RNA coronaviruses, MERS-CoV and SARS-CoV-2. Finally, we demonstrated that the modified RT-LAMP-coupled toehold reaction could detect MERS-CoV and SARS-CoV-2 with high sensitivity and specificity.

## 2. Results

### 2.1. RNA Toehold Switch Sensor Design and Screening

**Figure 1:**
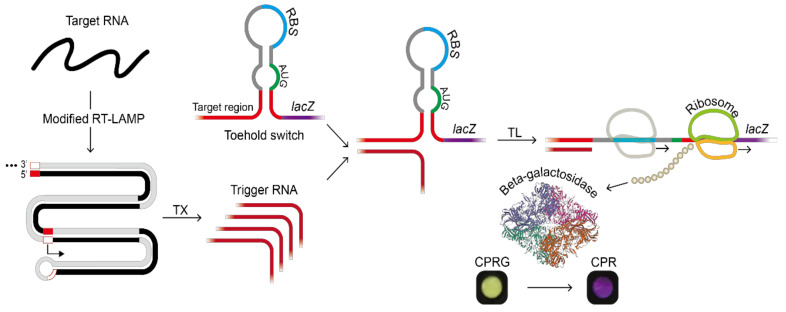
Target RNA detection scheme using the modified reverse transcription loop-mediated amplification (RT-LAMP) developed in this study and subsequent toehold reaction in a paper-based cell-free protein expression system. The reporter protein, beta-galactosidase, catalyzed the conversion of chlorophenol red-D-galactopyranoside (CPRG) to chlorophenol red (CPR). Beta-galactosidase structure was from PDB ID 6DRV, beta-galactosidase [17]. (Abbreviation: RBS, ribosome binding site; TL, in vitro translation; TX, in vitro transcription).

Due to the flexibility to design a pair of toehold switch and its cognate trigger RNA, the toehold reaction has been widely used as an RNA recognition-based detection system for pathogen identification and microbiome profiling [12,13,14]. The toehold switch contains a stem structure around the start codon (Figure 1) [15,16]. Once a single-stranded, 5′ end of toehold switch is bound with a trigger RNA, it untwists the stem structure of the downstream of the start codon by branch migration. This migration helps to alleviate the secondary structure around the start codon, leading to translation initiation.

**Figure 2:**
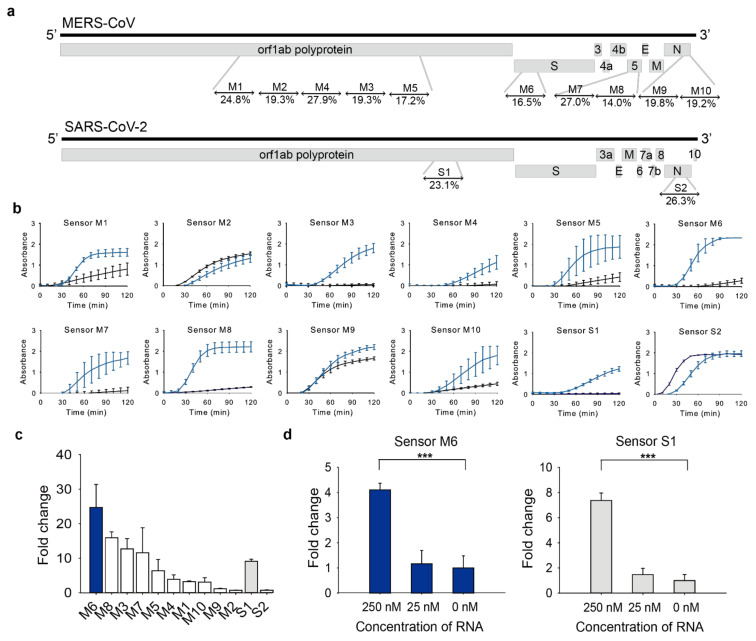
Toehold switch sensor screening. (**a**) Target regions and normalized ensemble defect (%) of each toehold switch sensor were indicated. (**b**) Absorbance changes at 570 nm of toehold reaction with or without trigger RNA in a cell-free system on a paper-disc. Blue line: toehold reaction with trigger RNA (on-state), Black line: toehold reaction without trigger RNA (off-state). (**c**) Fold changes of toehold switch sensors in Figure 2b at 60 min. (**d**) Sensitivity of sensor M6 and S1 with different concentrations of trigger RNA. Fold change was calculated as the toehold reaction with trigger RNA divided by that without trigger at 60 min. Two-tailed student’s test; *** *p* < 0.001; Error bars represent ± s.d., *n* = 3.

The toehold switches and trigger sequences were designed via NUPACK, a software that designs the sequence of interacting nucleic acids to have a designated secondary structure [18]. The toehold switch design in this study is based on the series B sensor from the previous publication [12]. We first used subsections of the entire MERS-CoV genome and SARS-CoV-2 genome as input sequences and selected the target regions with small normalized ensemble defects (NED, %), which implies an average percentage of incorrectly paired nucleotides at equilibrium over the target secondary structure (Figure 2a and Table S1).

As a reporter, we used *lacZ* gene encoding β-galactosidase that hydrolyzes chlorophenol red-D-galactopyranoside (CPRG, colored yellow) into chlorophenol red (CPR, colored purple). In this way, only when the target RNA sequence exists, the translation of the *lacZ* gene can be triggered. The resulting LacZ enzyme converts CPRG to CPR and the color change on a paper can be monitored through naked eyes (Figure 1) and quantified by measuring absorbance at 570 nm. Color change as Figure 1 was observed when there was a statistically significant difference between samples and negative controls.

The designed toehold switch sensors were tested with (on-state) or without (off-state) trigger RNA. Toehold switch sensors that show high on/off fold-change, that is, high absorbance at the on-state but low absorbance at the off-state, were selected for further experiments. Among the sensors tested, sensor M6 for MERS-CoV and sensor S1 for SARS-CoV-2 were selected for downstream tests as they showed the low absorbance in the off-state (Figure 2b) and the large fold change (Figure 2c). The sensors with low absorbance in the off-state are particularly useful because they can differentiate positives from negatives clearly with low backgrounds.

### 2.2. Sensitivity of Toehold Switch Sensors

Next, we evaluated the sensitivity of sensors, M6 and S1. After testing a series of target RNA concentrations, we found that the toehold reaction was initiated with as little as 250 nM of target RNA for both sensor M6 and S1 (Figure 2d). However, it was insufficient to detect the viral RNA, considering that the average viral load of MERS-CoV and SARS-CoV-2; 1.9 × $10^{4}$ copies/mL (31.6 aM) in an upper respiratory tract sample (URT) and 5 × $10^{6}$ copies/mL (8.3 fM) in a lower respiratory tract sample (LRT) for MERS-CoV [19] and 7.99 × $10^{4}$ copies/mL (133 aM) in a URT sample and 7.52 × $10^{5}$ copies/mL (1.25 fM) in an LRT sample for SARS-CoV-2 [20].

To enhance the detection limit, we introduced nucleic acid amplification technology. One of the isothermal amplification methods, NASBA, was commonly used to amplify RNA and the compatibility with the toehold reaction was already demonstrated [12]. However, the detection limit of NASBA-coupled toehold reaction was reported around a few femtomolar [12], which could be marginal for some samples. Therefore, we sought to explore another isothermal amplification method for the toehold reaction.

### 2.3. Connecting RT-LAMP with RNA Toehold Reaction

Reverse transcription loop-mediated isothermal amplification (RT-LAMP) is a rapid, one-step and straightforward isothermal amplification method [21]. Despite several advantages, RT-LAMP cannot be directly coupled to the toehold reaction because the amplified RT-LAMP product is in the form of DNA. To unfold the toehold switch sensor, its cognate trigger that contains the target sequence should be in the form of RNA. To use the RT-LAMP as an amplification method, we had to find a way to transcribe the RT-LAMP amplicon.

In order to generate transcripts from the amplified DNA, we used T7 RNA polymerase and its promoter, which have been often used for such conversion from DNA to RNA [9,12]. We first investigated insertion sites for the T7 promoter, from which the T7 RNA polymerization occurs. Since the T7 promoter sequence’s improper addition to an RT-LAMP primer could prevent the amplification, we carefully selected the insertion site in the RT-LAMP primer.

**Figure 3:**
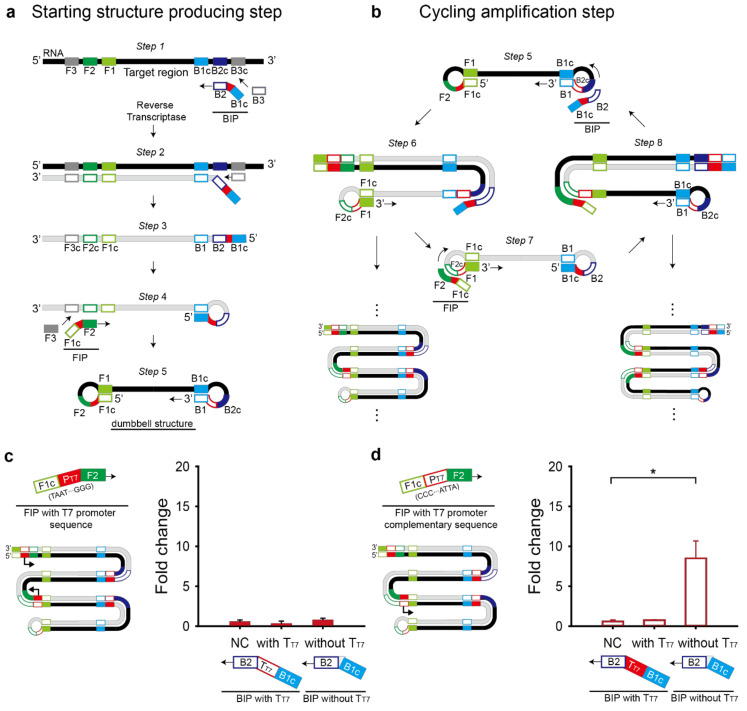
Schematic illustration of the modified RT-LAMP reaction consists of a starting-structure-producing step (**a**) and a cycling amplification step (**b**). (**c**) T7 promoter (P_T7_) and complementary T7 terminator (T_T7_) were inserted into inner primers, FIP and BIP, respectively. With different combinations of the modified primers, RT-LAMP and the subsequent toehold reaction in the paper-based cell-free system were performed. Fold change was calculated as the absorbance value with trigger RNA divided by that without the trigger RNA. (**d**) Complementary T7 promoter (P_T7_) and T7 terminator (T_T7_) were inserted into inner primers, FIP and BIP, respectively. Sensor M6 and its target RNA (20 pM) were used and the RT-LAMP was done without loop primers. For the fold change calculation, the absorbance value was obtained with 60-min RT-LAMP and 60-min toehold reaction. For the negative control (NC), the RT-LAMP reaction product with no target RNA was used. Two-tailed student’s test; * *p* < 0.05; Error bars represent ± s.d., *n* = 3.

The RT-LAMP reaction consists of a starting-structure-producing step and a cycling amplification step (Figure 3a,b). The starting structure is a dumbbell-like structure (Step 5 in Figure 3a), from which the cycling amplification can proceed. In the cycling amplification step, both starting and intermediate structures contain the self-primed DNA region, which elongates from 5′ to 3′ direction by a strand-displacing polymerase, called Bst DNA polymerase and the loop region, which recruits additional hybridization primers. The two features contribute to the target region amplification [21,22].

When the T7 promoter and T7 terminator are inserted at the 5′ end of outer primers (F3 and B3), the T7 sequences will be excluded from the dumbbell structure. When the T7 promoter and T7 terminator are inserted at the 3′ end of the outer primers, strand displacement cannot occur. Also, the addition of the T7 promoter and T7 terminator at each the 3′ or 5′ ends of the inner primers (FIP and BIP) will prevent self-primed elongation as illustrated in step 4 to 5 or step 6 to 7 when added at the 3′ or 5′ end of FIP, respectively and in step 1 to 2 or step 5 to 6 when added at the 3′ or 5′ end of BIP, respectively (Figure 3a,b).

Another option for the T7 promoter and T7 terminator is between F1c and F2 in the FIP primer and B1c and B2 in the BIP primer, respectively. First, the T7 promoter sequence was added to the FIP primer with direct or complementary sequence (Figure 3c,d). T7 promoter’s orientation affected the fold change of the toehold reaction. Only the FIP primer with the T7 promoter complementary sequence drove transcription of RT-LAMP amplicons containing the target region, showing the apparent fold change of the toehold reaction (Figure 3d). Interestingly, when we placed the T7 terminator in the BIP primer to enhance the transcriptional termination efficiency, no meaningful fold change was observed regardless of the T7 promoter direction (Figure 3c,d). Based on the result, we chose the FIP primer with the T7 promoter complementary sequence and the BIP primer without the T7 terminator for further experiments.

### 2.4. Sensitivity of the Modified RT-LAMP-Coupled Toehold Reaction on Contrived Samples

Loop primers that hybridize to the stem-loop regions of RT-LAMP intermediate products are known to accelerate the amplification reaction further [23]. We introduced the loop primers to improve the RT-LAMP sensitivity. The Loop F and B primers can be located between F1c and F2c and between B1c and B2c, respectively (Figure S1). Loop primers were designed for the sensor M6 and S1 (Table S2).

**Figure 4:**
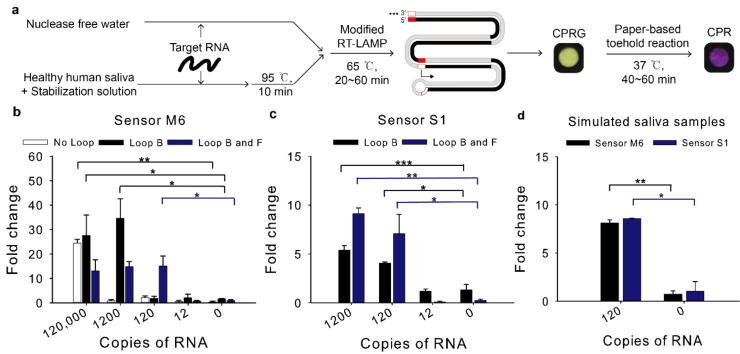
Detection limit of the modified RT-LAMP-coupled toehold reaction on contrived samples. The modified RT-LAMP is performed for 60 min with different combinations of loop primers (Loop B and Loop F). The resulting RT-LAMP product was applied to the subsequent toehold reaction. Fold change was calculated as the absorbance value from the toehold reaction with trigger RNA divided by that without the trigger RNA at 60 min. (**a**) Schematic illustration of the modified RT-LAMP and the subsequent toehold reaction. The paper assay image was the result of Figure 4d for sensor M6. (**b**) Effect of loop primers to improve detection limit of sensor M6. (**c**) Effect of different combinations of loop primers to detection limit of sensor S1. (**d**) Compatibility of human saliva with RT-LAMP-coupled toehold reaction. Loop B and Loop F were used for sensor M6 and S1. Two-tailed student’s test; * *p* < 0.05, ** *p* < 0.01, *** *p* < 0.001; Error bars represent ± s.d., *n* = 3.

The detection limit of the modified RT-LAMP-coupled toehold reaction was tested by using in vitro transcribed target RNA spiked in either water unless mentioned otherwise or saliva (Figure 4a).

For sensor M6, when the Loop primer B was used, the detection limit was dramatically enhanced compared to the assay with no loop primers, reaching 1200 copies per reaction (120 copies/μL, 200 aM) (Figure 4b). When both Loop B and F primers were used, the reaction detected as few as 120 copies per reaction (12 copies/μL, 20 aM), which would be sufficient sensitivity to detect MERS-CoV virus in clinical samples [19].

For sensor S1, when the RT-LAMP resultant using the Loop B and F primers were employed for the toehold reaction, 120 copies per reaction (12 copies/μL, 20 aM) induced the toehold reaction (Figure 4c), implying that SARS-CoV-2 viruses in clinical samples can be detected with the modified RT-LAMP-coupled toehold reaction developed in this study [20].

To determine this assay is compatible with possible inhibitors of human samples, synthetic RNA spiked into human saliva was used as proxy clinical samples. Nasopharyngeal swab (NP swab) is a standard collection sample for diagnosis of respiratory infection. However, the NP swab is relatively invasive [24]. As an alternative, saliva is selected as a non-invasive sample and it has the viral load sufficient for SARS-CoV-2 detection [25]. Heating unextracted diagnostic samples to obliterate nucleases (HUDSON) has been used to inactivate ribonucleases and lyse viral particles in saliva samples using tris(2-carboxyethyl)phosphine hydrochloride (TCEP-HCl), ethylenediaminetetraacetic acid (EDTA) and heat treatment [26].

It is known that 10% of human saliva samples are acidic, which can affect the pH in LAMP reaction [27]. The toehold switch sensor is one of riboswitches of which structure can change depending on the pH [28]. Human saliva could relieve the sequestered start codon in the toehold switch sensor and translation could be activated even without trigger RNA. To remove the possibility of false positives, we used saliva stabilization solution similar to the HUDSON protocol but different in point of using basic solution to increase the pH of saliva samples [27].

Saliva samples from healthy humans were spiked with MERS-CoV or SARS-CoV-2 RNA at a 1:5 ratio and they were mixed with the previous 2X optimized saliva stabilization solution at a 1:1 ratio [27]. Then, they were incubated at 95 °C for 10 min. Heat inactivated simulated saliva samples were then used for RT-LAMP-coupled toehold reaction. The reaction detected 120 copies in diluted saliva samples and showed no false positives for sensor M6 and S1 (Figure 4d).

### 2.5. Reduction of the Overall Turnaround Time

**Figure 5:**
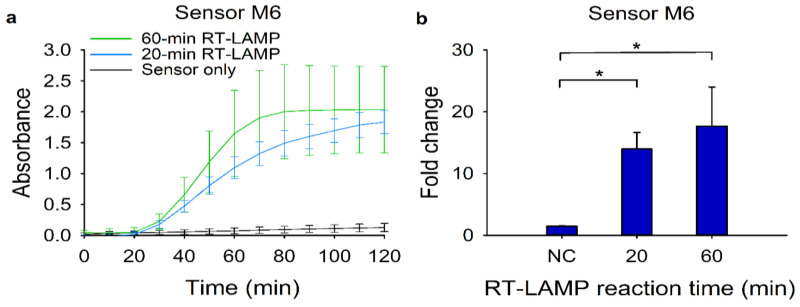
Reduction of the overall reaction time for the modified RT-LAMP and the subsequent toehold reaction. For the reduction of overall reaction time, 120 copies of target RNA for sensor M6 were used. (**a**) Absorbance (570 nm) changes of the toehold reaction with or without trigger RNA using sensor M6. Blue and green lines indicate the toehold reaction after 20-min and 60-min RT-LAMP reactions, respectively, in the presence of target RNA. The black line denotes the same reaction for 60 min in the absence of target RNA. (**b**) Fold change was calculated as the absorbance values from toehold reaction with trigger RNA divided by that without the trigger RNA at 40 min. For the negative control (NC), the RT-LAMP reaction product with no target RNA was used. Two-tailed student’s test; * *p* < 0.05; Error bars represent ± s.d., *n* = 3.

We then investigated the feasibility of the modified RT-LAMP-coupled toehold reaction as a rapid diagnostic test. To reduce the overall turnaround time, we first cut down the RT-LAMP reaction time from 60 min to 20 min. Using the RT-LAMP amplicons, we monitored the absorbance change of the toehold reactions (Figure 5a). We observed that a 40-min toehold reaction would be enough to discriminate the target signal from the background. Moreover, the 20-min RT-LAMP reaction had a similar fold change with a 60-min RT-LAMP reaction (Figure 5b). Collectively, the 10-min heat inactivation and the 20-min RT-LAMP followed by the 40-min toehold reaction time would be sufficient to detect 120 copies of synthetic MERS-CoV RNA.

### 2.6. Specificity of the Modified RT-LAMP-Coupled Toehold Reaction

**Figure 6:**
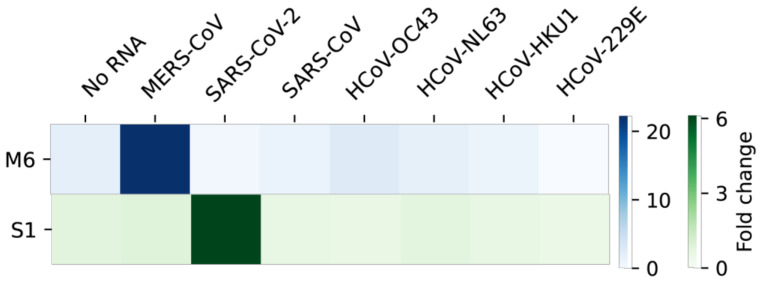
Specificity of the modified RT-LAMP-coupled toehold reaction against other coronaviruses. Sensor M6 for MERS-CoV and sensor S1 for SARS-CoV-2 were tested with several coronaviral RNAs. For the specificity test, 12,000 copies of target RNA were used for RT-LAMP. Fold change was calculated by taking absorbance values at 60 min using a 60-min RT-LAMP reaction product. Colors in the heatmap indicate the mean value (*n* = 3) of fold changes of the toehold reaction and the scales are given on the right.

Since the coronaviruses share substantial sequence similarities, we tested the MERS-CoV or SARS-CoV-2 sensors for possible cross-reactivity against other coronaviruses, including SARS-CoV, HCoV-OC43, HCoV-NL63, HCoV-HKU1 and HCoV-229E. To do so, we first aligned each viral sequence homologous to the target region of MERS-CoV (Figure S2) or SARS-CoV-2 (Figure S3). Each homologous region to the target was synthesized, transcribed into RNA and tested against the toehold sensors (Figure 6). Not surprisingly, the sensor M6 detected only its target, MERS-CoV, with high specificity and sensor S1 exclusively identified its target, SARS-CoV-2, indicating no cross-reactivity of the modified RT-LAMP-coupled toehold reaction against other non-target coronaviruses.

## 3. Discussion

RNA toehold switch sensors are robust molecular switches that can be used to detect RNA molecules in a sequence-specific manner. This flexible switch had been combined with a paper-based cell-free molecular diagnostic platform to detect various target nucleic acids [12,13,14]. However, to enable the sensitive detection of nucleic acids, toehold reactions require an amplification step that multiplies target RNA. The isothermal nucleic acid amplification method has been often applied to this molecular diagnosis to enhance the detection limit. The previously adapted method was NASBA, which takes 1–3 h for the amplification and requires multiple preparation steps to acquire reaction mixtures. As an alternative, we introduced RT-LAMP as an isothermal amplification method for the toehold reaction. RT-LAMP recently emerged as a promising molecular diagnosis method due to its short reaction time and high amplification potential. However, to trigger the toehold reaction, the RT-LAMP amplicon’s output product must be transcribed into RNA. To address the challenge, we strategically modified RT-LAMP primers to include the T7 promoter sequence in the RT-LAMP amplicon and found that the transcript of the RT-LAMP amplicon was successfully untwisted the toehold switch sensor, enabling the LacZ-mediated colorimetric detection.

Interestingly, the addition of a T7 terminator, which is typically known to improve the termination efficiency, was not helpful in this case. Possible explanation is due to the failure of RT-LAMP. When we analyzed the RT-LAMP product, a typical ladder-like amplicon pattern observed in the agarose gel was not seen with the T7 terminator-inserted case, indicating the failure of RT-LAMP (Figure S4). The RT-LAMP was also failed with the forward T7 promoter addition to the FIP (Figure 3a and Figure S4). Considering the strong RT-LAMP amplification with the complementary T7 promoter addition (Figure 3b and Figure S4), this result implies that the success of the modified RT-LAMP depends on flanking sequences around the insertion sequence in the primer and thus locus and direction of the insertion sequence should be carefully tested and selected.

The modified RT-LAMP-coupled toehold reaction developed in this study achieved high sensitivity. Notably, for both MERS-CoV and SARS-CoV-2 target RNA, we detected as few as 120 copies per reaction (12 copies/μL, 20 aM), the highest sensitivity reported using a toehold switch sensor. When NASBA was used as an amplification step for the toehold reaction, the detection limit was 3 fM of Zika viral RNA [12]. In another report, when recombinase polymerase amplification (RPA) was connected to the toehold reaction, the detection limit was around 250 aM [14]. The sensitivity we achieved in this study indicates the modified RT-LAMP is an appropriate and compatible isothermal amplification method for the toehold reaction.

Moreover, the modified RT-LAMP that we demonstrated in this study can amplify target RNA in a relatively short period of time. Usually, LAMP is a fast isothermal amplification method that requires 20–60 min [29]. NASBA required 120 min to amplify 3 fM of the Zika virus [12]. RPA took 120–160 min to amplify target RNA [14]. Compared to these isothermal amplifications, the modified RT-LAMP demonstrated in this study amplified the 120 copies (12 copies/μL, 20 aM) of target RNA in 20 min, allowing the reduction of overall reaction time to 70 min.

In summary, we developed the modified RT-LAMP that can amplify target RNA and convert the resulting amplicon into RNA molecules. This new amplification method connected to the toehold reaction based on a paper-based cell-free expression system, which enabled colorimetric detection of MERS-CoV and SARS-CoV-2. This modified RT-LAMP-coupled toehold reaction showed high sensitivity (120 copies per reaction), high selectivity against 7 other coronaviruses and short turnaround time (70 min). We believe the modified RT-LAMP toehold reaction developed in this study would be useful to detect not only coronaviruses but also any organisms that contains RNA in a fast, sensitive, selective and colorimetric manner.

## 4. Materials and Methods

### 4.1. Toehold Switch Design

The genome sequences of MERS-CoV and SARS-CoV-2 were obtained from GenBank (Accession number: JX869059 for MERS-CoV and NC_045512 for SARS-CoV-2). For MERS-CoV, we first divided the genome sequence into 30 sections and ran NUPACK to choose target sequences and toehold switches [18]. The toehold switch structure was based on the series B sensor from the previous publication [12]. The toehold switch should have the toehold switch B structure and form a duplex structure with its trigger RNA. Candidate pairs of target and toehold switch with small normalized ensemble defect (%) were selected for the test (sensor M1–M3, M5, M6 and M8–M10). In addition, we also specifically targeted *orf1ab* and *upE* regions, which are commonly used targets for conventional RT-PCR probes [1]. Following the same sensor selection procedure, we obtained sensor M4 and M7. We selected toehold sensors for SARS-CoV-2 through the same procedure and they were from *orf1ab* and *N* gene regions for S1 and S2, respectively. Unwanted in-frame stop codons at the N terminus of the *lacZ* gene were discarded. DNA oligonucleotides (Integrated DNA Technologies, Coralville, IA, USA) containing toehold switch sequences were assembled into a vector that contains the *lacZ* reporter gene (Addgene, plasmid number: 75006, Watertown, MA, USA) using Gibson assembly (NEB, E2621L, Ipswich, MA, USA).

### 4.2. Paper Preparation

We followed the previously described paper preparation protocol [9]. Briefly, filter paper (Whatman, 1442-042, Little Chalfont, Bucks, UK) was autoclaved for 90 min at 121 °C and blocked in 5% BSA (Sigma-Aldrich, A2153, St. Louis, MO, USA) overnight. The blocked paper was rinsed three times with nuclease-free water and dried at 65 °C in a drying oven. The paper was punched into discs using a Biopsy Punch with Plunger (Inner Diameter 2 mm, Ted pella, 15110-20) and placed into a black, clear-bottom 384-well plate (Corning, 3540, Corning, NY, USA).

### 4.3. Cell-Free Reaction

Cell-free reaction contains components necessary for in vitro transcription and translation [30]. Cell-free reaction consisted of Solution A (40%), Solution B (30%) of in vitro protein synthesis kit (NEB, E6800), CPRG (Sigma-Aldrich, 59767, 0.6 mg/mL; 4%), RNase inhibitor (NEB, M0314L; 2%), DNA encoding a toehold switch (linear DNA, 100 ng/μL except for sensor S1, 90 ng/μL; 8%) and trigger RNA amplified by LAMP or nuclease-free water (16%). The linear DNA concentration was measured using the Qubit 4 fluorometer (ThermoFisher Scientific, Waltham, MA, USA). Cell-free reaction mixture (1.8 μL) was loaded onto a paper disc and absorbance at 570 nm was measured at 37 °C using a plate reader (Molecular Devices, SpectraMax ABS, San Jose, CA, USA).

### 4.4. Synthetic Target RNA Synthesis and Toehold Switch Sensor Screening

Template DNAs that include target RNA regions with 100 bp-extra bases at each end and T7 promoter sequence were synthesized (Integrated DNA Technologies). The template DNAs were in vitro transcribed into RNA. For the in vitro transcription, 200~1000 ng of target DNA, 2 μL 10X reaction buffer, 1 μL DTT (Bioneer, E3121L, 100 mM, Daejeon, Korea), 0.8 μL NTPs (NEB, M0466L, 1 mM for each NTP), 0.5 μL RNase Inhibitor (NEB, 0314L), 2 μL T7 RNA polymerase (NEB, M0251L) and RNase-free water up-to 20 μL were mixed. The mixture was incubated at 37 °C for 16 h, then treated with 1 μL of DNase I (NEB, M0303L) at 37 °C for 30 min. The resulting RNA was purified with the Riboclear™(plus!) RNA kit (GeneAll, 313–150, Seoul, Korea) and used as target RNA. The target RNA was quantified (ThermoFisher Scientific, Qubit 4 Fluorometer) and diluted in nuclease-free water as needed.

For the initial screening of toehold switch sensors, M1 to M10, for MERS-CoV, a high concentration of trigger RNA (2500 nM) that was obtained via in vitro transcription was directly used to induce the toehold-regulated expression of *lacZ* in the cell-free reaction. Meanwhile, sensors for SARS-CoV-2 were directly reacted with transcripts of the RT-LAMP amplicon yielded from 200 aM (sensor S1) or 1 fM (sensor S2) of initial target RNA in the cell-free reaction. To obtain the fold change, we first subtracted background absorbance that does not contain sensor DNA and trigger RNA from each absorbance value of the cell-free reaction with or without trigger RNA. At this stage, any negative value was adjusted to zero. Then, the fold change was calculated as the adjusted absorbance of the sensor with trigger RNA divided by that without the trigger RNA.

### 4.5. Reverse Transcription Loop-Mediated Isothermal Amplification (RT-LAMP)

The RT-LAMP primers were designed using Primer Explorer v.5 software (https://primerexplorer.jp/e/, accessed on 20 April 2020). The parameter of F1-B1c distance was modified from 0–100 to 36–100 to locate a target region of the toehold switch sensor between F1 and B1c. For the RT-LAMP reaction, WarmStart LAMP Kit 2X Master Mix (NEB, E1700; 50%), which contains a strand displacement DNA polymerase and reverse transcriptase in an optimized buffer, 10X Primer Mix (16 μM of each FIP and BIP primers, 2 μM of each F3 and B3 primers and 4 μM of each Loop F or Loop B primers; 10%), target RNA (40%) were assembled at room temperature according to the manufacturer’s recommendation. The reaction mix was incubated at 65 °C for 60 min in a heat block, then heat-inactivated at 80 °C for 5 min. The reaction products were directly used in the subsequent cell-free reaction.

### 4.6. Saliva Inactivation with Heat and Stabilization Solution

Stabilization solution was prepared with TCEP-HCl (Sigma-Aldrich, 75259), EDTA (ThermoFisher Scientific, AM9260G), NaOH (Sigma-Aldrich, 72068), Proteinase K (Geneall, 106–101) and Nuclease free water. The final concentration of 2X stabilization solution was 5 mM for TCEP-HCl, 2 mM for EDTA, 29 mM for NaOH and 100 μg/mL for Proteinase K. 6 μL of stabilization solution was added to 1 μL Saliva (Lee Biosolution, 991-05-P, St. Louis, MO, USA) mixed with 5 μL synthetic RNA in nuclease-free water. It was heated at 95 °C for 10-min and 10 μL of it was put into LAMP reaction.

### 4.7. Specificity Test

We investigated the cross-reactivity of the toehold sensors against other closely related coronaviruses. Each homologous region of the other coronaviruses to the target region for sensor M6 or S1 was first synthesized and a T7 promoter was included in the upstream of each homologous region (Figures S2 and S3). The synthesized DNA was in vitro transcribed, as described above. The resulting RNA containing target homologous regions were used for the RT-LAMP. For each sensor test, 12,000 copies of target RNA were used. RT-LAMP with the loop primers was performed as described above. The RT-LAMP amplicon was directly used in the cell-free reaction.

## Supplementary Materials

Supplementary Materials can be found at https://www.mdpi.com/1422-0067/22/4/1772/s1. Table S1. Sequences of toehold switch sensors and target regions; Table S2. List of RT-LAMP primer sequences; Figure S1. Schematic representation of Loop F and Loop B primers in the LAMP dumbbell-like structure; Figure S2. Sensor M6’s target RNA and its upstream and downstream flanking sequences in the *S* gene of MERS-CoV; Figure S3. Sensor S1’s target RNA and its upstream and downstream flanking sequences in the *orf1ab* gene of SARS-CoV-2; Figure S4. Gel electrophoresis of the modified RT-LAMP products.

## Abbreviations

RT-LAMP	Reverse transcription loop-mediated isothermal amplification
rRT-PCR	Real-time reverse transcription polymerase chain reaction
NASBA	Nucleic acid sequence-based amplification
RBS	Ribosome binding site
CPRG	Chlorophenol red-D-galactopyranoside
CPR	Chlorophenol red
URT	Upper respiratory tract sample
LRT	Lower respiratory tract sample
FIP	Forward inner primer
BIP	Backward inner primer
RPA	Recombinase polymerase amplification
TX	in vitro transcription
TL	in vitro translation
