# Supplementary material for: Detection of Coronaviruses Using RNA Toehold Switch Sensors
Source: Int J Mol Sci. 2021 Feb 10;22(4):1772. doi: 10.3390/ijms22041772 (PMC7916667; doi:10.3390/ijms22041772)
Supplement: Supplementary file 1 [file ijms-22-01772-s001.pdf]

Supplementary Information

Detection of Coronaviruses using RNA Toehold Switch Sensors

Soan Park and Jeong Wook Lee

Table S1. Sequences of toehold switch sensors and target regions.

Target region	Location in genome	Normalized ensemble defect (%)	Sensor name	Sensor sequence
AGUUUCCAGGACGCUGCCUCUAAUAUCUUUGUUAUU	9756 (*orf1ab* gene)	24.8	M1	**TAATACGACTCACTATAGGG**AATAACAAAGATATTAGAGGCAGCGTCCTGGAAACTGGACTTTAGAACAGAGGAGATAAAGATG***AGTTTCCAGGAC***AACCTGGCGGCAGCGCAAAAGATGCGTAAA
GCAAACAUAGUCUACGAGCCCACUACUCCCAUUUCG	11178 (*orf1ab* gene)	19.3	M2	**TAATACGACTCACTATAGGG**CGAAATGGGAGTAGTGGGCTCGTAGACTATGTTTGCGGACTTTAGAACAGAGGAGATAAAGATG***GCAAACATAGTC***AACCTGGCGGCAGCGCAAAAGATGCGTAAA
ACUUAUGCCAUCUUUGCUUACUCACCACAGCUUACA	11511 (*orf1ab* gene)	19.3	M3	**TAATACGACTCACTATAGGG**TGTAAGCTGTGGTGAGTAAGCAAAGATGGCATAAGTGGACTTTAGAACAGAGGAGATAAAGATG***ACTTATGCCATC***AACCTGGCGGCAGCGCAAAAGATGCGTAAA
ACUACUCCCAUUUCGUCAGCGCUGAUUGCAGUUGCA	11199 (*orf1ab* gene)	27.9	M4	**TAATACGACTCACTATAGGG**TGCAACTGCAATCAGCGCTGACGAAATGGGAGTAGTGGACTTTAGAACAGAGGAGATAAAGATG***ACTACTCCCATT***AACCTGGCGGCAGCGCAAAAGATGCGTAAA
AGUUCAUGUCUACUCCCAAUGCCAUCUUUAUUUCUG	19953 (*orf1ab* gene)	17.2	M5	**TAATACGACTCACTATAGGG**CAGAAATAAAGATGGCATTGGGAGTAGACATGAACTGGACTTTAGAACAGAGGAGATAAAGATG***AGTTCATGTCTG***AACCTGGCGGCAGCGCAAAAGATGCGTAAA
AAAUGGGCCGCUUCUUCAAUCAUACUCUAGUUCUUU	21934 (*S* gene)	16.5	M6	**TAATACGACTCACTATAGGG**AAAGAACTAGAGTATGATTGAAGAAGCGGCCCATTTGGACTTTAGAACAGAGGAGATAAAGATG***AAATGGGCCGCA***AACCTGGCGGCAGCGCAAAAGATGCGTAAA
CUCGCUUAUCGUUUAAGCAGCUCUGCGCUACUAUGG	27499 (*orf5* gene)	27.0	M7	**TAATACGACTCACTATAGGG**CCATAGTAGCGCAGAGCTGCTTAAACGATAAGCGAGGGACTTTAGAACAGAGGAGATAAAGATG***CTCGCTTATCGA***AACCTGGCGGCAGCGCAAAAGATGCGTAAA
ACACACCAAACCAUUAUUUAUUAGAAACUUCGAUCA	27280 (*orf5* gene)	14.0	M8	**TAATACGACTCACTATAGGG**TGATCGAAGTTTCTAATAAATAATGGTTTGGTGTGTGGACTTTAGAACAGAGGAGATAAAGATG***ACACACCAAACA***AACCTGGCGGCAGCGCAAAAGATGCGTAAA
GAGCCAUUAAACUUGACCCAAAGAAUCCCAACUACA	29563 (*N* gene)	19.8	M9	**TAATACGACTCACTATAGGG**TGTAGTTGGGATTCTTTGGGTCAAGTTTAATGGCTCGGACTTTAGAACAGAGGAGATAAAGATG***GAGCCATTAAAC***AACCTGGCGGCAGCGCAAAAGATGCGTAAA
GCCAUUAAACUUGACCCAAAGAAUCCCAACUACAAU	29565 (*N* gene)	19.2	M10	**TAATACGACTCACTATAGGG**ATTGTAGTTGGGATTCTTTGGGTCAAGTTTAATGGCGGACTTTAGAACAGAGGAGATAAAGATG***GCCATTAAACTA***AACCTGGCGGCAGCGCAAAAGATGCGTAAA
AUGUCAUAUCAACAUCACAUAAAUUAGUCUUGUCUG	16334 (*orf1ab* gene)	23.1	S1	**TAATACGACTCACTATAGGG**CAGACAAGACTAATTTATGTGATGTTGATATGACATGGACTTTAGAACAGAGGAGATAAAGATG***ATGTCATATCAG***AACCTGGCGGCAGCGCAAAAGATGCGTAAA

AUGACAAAGAUCCAAAUUUCAAAGAUCAAGUCAUUU	29292 (*N* gene)	26.3	S2	**TAATACGACTCACTATAGGG**AAATGACTTGATCTTTGAAATTTGGATCTTTGTCATGGACTTTAGAACAGAGGAGATAAAGATG***ATGACAAAGATA***AACCTGGCGGCAGCGCAAAAGATGCGTAAA

The sequence of sensors consists of **T7 promoter** + target region (a + b) + conserved domain sequence + ***a part of target region*** (complementary sequence of stem region, b′) + conserved domain sequence. The conserved domain sequence was from the series B sensor from the previous publication [1].

Table S2. List of RT-LAMP primer sequences

**Sensor**†	**RT-LAMP primer**	**Sequence (5' to 3')**
M6	F3	ATTAGCCCATCTACCAGC
	B3	TGGCAAAAGAAGTATAGGAATT
	FIP with T7 promoter (5′ F1c-T7 promoter-F2 3′)	ACCATCTGAGAAATTACCAACTGAATAATACGACTCACTATAGGGGCTACTATACGAAAAATTTACCCT
	FIP with T7 promoter complementary sequence (5′ F2c-T7 promoter-F1 3′)	ACCATCTGAGAAATTACCAACTGAACCCTATAGTGAGTCGTATTAGCTACTATACGAAAAATTTACCCT
	BIP with T7 terminator complementary sequence (5′ B2c-T7 terminator-B1 3′)	CCGATGGATGTGGCACTTTACCAAAAAACCCCTCAAGACCCGTTTAGAGGCCCCAAGGGGTTATGCTACAGGACAATGATTTCCAGAG
	BIP with T7 terminator (5′ B1c-T7 terminator-B2 3′)	CCGATGGATGTGGCACTTTACTAGCATAACCCCTTGGGGCCTCTAAACGGGTCTTGAGGGGTTTTTTGCAGGACAATGATTTCCAGAG
	BIP without T7 terminator	CCGATGGATGTGGCACTTTACCAGCAGGACAATGATTTCC
	Loop F‡	GAACCCAGCATAAAAGC
	Loop B	TTTATTGTATTCTAGAGCCTCGCTC
S1	F3	TGGGGCTTGTGTTCTTTG
	B3	CTAATGGGTGGTTTATGTGATT
	FIP with T7 promoter complementary sequence (5′ F2c-T7 promoter-F1 3′)	TGGTCGTAACAGCATTTACAACATACCCTATAGTGAGTCGTATTAACAGACTTCATTAAGATGTGGT
	BIP without T7 terminator	TAATCCGTATGTTTGCAATGCTCCCATACCTCCTAAGTAAAGTTGAG
	Loop F‡	AGAATGGTCTACGTATGCAAGC
	Loop B	AGGTTGTGATGTCACAGATGTG
S2	F3	GTTCTTCGGAATGTCGCG
	B3	CGGTAAGGCTTGAGTTTCA
	FIP with T7 promoter complementary sequence (5′ F2c-T7 promoter-F1 3′)	CCAATTTGATGGCACCTGTGTCCCTATAGTGAGTCGTATTAATTGGCATGGAAGTCACAC
	BIP without T7 terminator	AAGCATATTGACGCATACAAAACATGCCTTCTTCTTTTTGTCCTT
	Loop F	GGTCAACCACGTTCCCGA
	Loop B	TCCCACCAACAGAGCCTAAA

†Target region of each sensor is amplified by RT-LAMP.

‡Loop F primer of M6 and S1 was obtained by changing the parameter condition from the default setting, Tm 60 °C to 51 °C and dimer check -3.5 to -4.5.

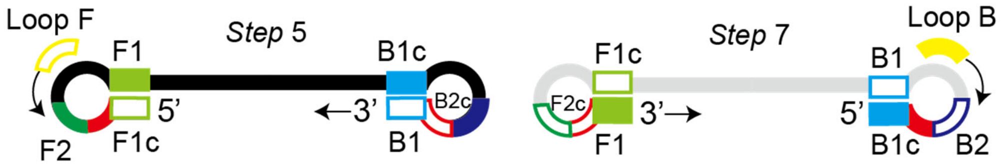

Figure S1. Schematic representation of Loop F and Loop B primers in the LAMP dumbbell-like structure

```
MERS-CoV     1    -TTGCTAATGGGTTTGTCGTCCGTATAGGAGCAGCTGCCAA-TTCCACTG
SARS-CoV     1    -TTGTCCGTGGTTGGGTTTTTGGTTCTACCATGAACAACA--AGTCACAG
SARS-CoV-2   1    -TAATAAGAGGCTGGATTTTTGGTACTACTTTAGATTCGA--AGACCCAG
HCoV-OC43    1    -TTATTAAAGATCGTGTAATGTATAGTGAGTTCCCTGCTAT-AACTATAG
HCoV-NL63    1    AACACTACTTTTGATTTTTTAAGTAATGCTTCTAGTTCTTTTGACTGTAT
HCoV-HKU1    1    -TGTATGTTAATAAAACTTTGTATAGTGAGTTTAGTACTAT-AGTTATAG

MERS-CoV     51   GCACTG-TTATTATTAGCCCATCTACCAG-CGCTACTATACGAAAAATTT
SARS-CoV     51   TCGGTGATTATTATTAACAATTCTACTAA-TGTTGTTATACGAGCATGTA
SARS-CoV-2   51   TCCCTACTTATTGTTAATAACGCTACTAA-TGTTGTTATTAAAGTCTGTG
HCoV-OC43    51   GTAGTACTT-TTGTAAATACATCCTATAG-TGTGGTAGTACAACCACGTA
HCoV-NL63    51   AGTTAATTTGTTATTTACAGAACAGTTAGGTGCGCCTTTGGGCATAACTA
HCoV-HKU1    51   GTAGTGTTT-TTATTAACAACTCTTATAC-TATTGTTGTTCAACCTCATA

MERS-CoV     101  ACCCTGCTTTT-------ATGCTGG----------------GTTCTTCAG
SARS-CoV     101  ACTTTGA-----------ATTGTG--------TGACAACCCTTTCT----
SARS-CoV-2   101  AATTTCA-----------ATTTTG--------TAATGATCCATTTT----
HCoV-OC43    101  CAATCAATTCAACACAGGATGGTGATAATAAATTACAAGGTCTTTTAGAG
HCoV-NL63    101  TATCTGGTGAA-------ACTGTG-----------CGTCTGCATTTATAT
HCoV-HKU1    101  ------------------ATGGTG------------------TTTTGGAG

MERS-CoV     151  TTGGTAATTTCTCAGATGGTAAAATGGGCCGCTTCTTCAATCATACTCTA
SARS-CoV     151  -TTGCTGTTTCTAAAC--CCA-TGGGTA-CACAGAC---ACATACTATGA
SARS-CoV-2   151  -TGGGTGTTTATTACC--ACA-AAAACAACAAAAGTTGGATGGAAAGTGA
HCoV-OC43    151  GTCTCTGTTTGCCAGT--ATAATATGTGCGAGTACCCACAAACGATTTGT
HCoV-NL63    151  AATGTAACTCGT-ACT---TTTTATGTGCCAGCAGCTT-ATAAACTTACT
HCoV-HKU1    151  ATTACAGCTTGTCAAT--ACACTATGTGTGAGTATCCTCATACTATTTGT

MERS-CoV     201  GTTCTTTTGCCCGATGGATGTGGCACTTTACTTAGAGCTTTTTATTGTAT
SARS-CoV     201  TATTCGA-----------TAATGCA-TTTAATTGCA-CTTTCGAGTACAT
SARS-CoV-2   201  GTTCAGAG---TTTATTCTAGTGCG-AATAATTGCA-CTTTTGAATATGT
HCoV-OC43    201  CATCCTAACCTGGGTAATCATCGCA-AAGAACTATGGCATTTGGATACAG
HCoV-NL63    201  AAACTTAGTGTTAAATGTTACTTTA-ACTATTCCTGTGTTTTTAGTGT-T
HCoV-HKU1    201  AAATCTAA---AGGTAGTTCTCGTA-ATGAATCTTGGCATTTTGATAAAT

MERS-CoV     251  -TCTAGAGCCTCGCTCTGGAAATCATTGTCCTGCTGGCAATTCCTATACT
SARS-CoV     251  ATCTGATGCCTTTTCGCTTGATGTTTCAGAAAA-GTCAGGTAATTTTAAA
SARS-CoV-2   251  CTCTCAGCCTTTTCTTATGGACCTTGAAGGAAA-ACAGGGTAATTTCAAA
HCoV-OC43    251  GTGTTGTTTCCTGTTTATATAAGCGTAATTTCACATATGATGTGAATGCT
HCoV-NL63    251  GTCAACGCCACCGTTACTGTGAATGTCACCACA--CATAATGGCCGTGTA
HCoV-HKU1    251  CTGAACCTTTGTGTCTGTTCAAGAAAAATTTTACTTATAATGTTTCTACA

MERS-CoV     301  TCTTTTGCCACTTATCACACTCCTGCAACAGATTGTTCTGATGGCAATTA
SARS-CoV     301  CACTTACGAGAGTTTGTGTTTAAAAATAAAGATGGGTTTCTCTATGTTTA
SARS-CoV-2   301  AATCTTAGGGAATTTGTGTTTAAGAATATTGATGGTTATTTTAAAATATA
HCoV-OC43    301  GATTATTTGTATTTTCATTTTTATCAAGAAGGTGGTACTTTTTATGCATA
HCoV-NL63    301  GTTAACTACACTGTTTGTGATGATTGTAATGGTTATACTGATAACATATT
HCoV-HKU1    301  GATTGGTTGTATTTTCATTTTTATCAAGAACGTGGCACTTTTTATGCTTA

MERS-CoV     351  CAATCGTAATGCCAGTCTGAACTCTTTTAAGGAGTATTTTAATTTACGTA
SARS-CoV     351  TAAGGGCTATCA------A---CCTATAGATGTAGTTCGTGATCTACCTT
SARS-CoV-2   351  TTCTAAGCACAC------G---CCTATTAATTTAGTGCGTGATCTCCCTC
HCoV-OC43    351  TTTTACAGACACTGGTGTT---GTTACTAAGTTTTTGTTTAATGTTTATT
HCoV-NL63    351  TTCTGTTCAACAGGATGGCCGCATTCCTAATGGTTTCCCT--TTTAATAA
HCoV-HKU1    351  TTATGCTGATTCTGGCATG---CCTACTACTTTTTTATTTAGTTTGTATC
```

Figure S2. Sensor M6's target RNA and its upstream and downstream flanking sequences in the *S* gene of MERS-CoV. Homology regions to this sequence from other coronaviruses including SARS-CoV, SARS-CoV-2, HCoV-OC43, HCoV-NL63, and HCoV-HKU1 were aligned for sequence comparison. The red box indicates the homologous sequences of the target region of sensor M6. The blue box denotes the homologous sequences of its

upstream and downstream flanking sequences that were synthesized for the cross-reactivity test. HCoV-229E sequence was excluded from the alignment; it showed many mismatches and gaps.

```
SARS-CoV-2  1    - - - - - - - - - G C T G T T G G G G C T T G T G T T C T T T G C A A T T C A C A G A C T T C A T T
SARS-CoV    1    G T C T T G C A G G C T G T A G G T G C T T G T G T A T T G T G C A A T T C A C A G A C T T C A C T
MERS-CoV    1    A C T T T G C A G G C T G T C G G T T C A T G C G T T G T A T G C C A T T C A C A G A C T T C C C T
HCoV-229E   1    G T A T T A C A A G C T G C T G G T C T T T G T G T A G T A T G T G G T T C T C A A A C A G T T C T
HCoV-OC43   1    G T T A T G C A G A G T G T T G G A G C T T G C G T G G T C T G C T C T T C T C A A A C A T C A T T
HCoV-NL63   1    A T A T T G C A A G C T G C T G G T T T A T G T G T T G T T T G T G G T T C A C A A A C T G T A C T
HCoV-HKU1   1    G T G A T G C A G A G T G T A G G T G C A T G C G T T G T T T G T T C A T C A C A A A C T T C T T T

SARS-CoV-2  51   A A G A T G T G G T G C T T G C A T A C G T A G A C C A T T C T T A T G T T G T A A A T G C T G T T
SARS-CoV    51   T C G T T G C G G T G C C T G T A T T A G G A G A C C A T T C C T A T G T T G C A A G T G C T G C T
MERS-CoV    51   A C G C T G T G G G A C A T G C A T C C G T A G A C C A T T T C T C T G C T G T A A A T G C T G C T
HCoV-229E   51   A A G A T G C G G T G A T T G T T T A C G C A G A C C G A T G T T G T G C A C T A A G T G C G C C T
HCoV-OC43   51   A C G T T G T G G C A G T T G C A T C A G A A A G C C T C T T C T T T G C T G C A A G T G T T G T T
HCoV-NL63   51   T C G T T G T G G T G A T T G T C T G C G T A A G C C T A T G T T G T G C A C T A A A T G C G C A T
HCoV-HKU1   51   G C G T T G T G G C A G T T G T A T A C G T A A G C C T T T G T T A T G T T G T A A A T G T T G T T

SARS-CoV-2  101  A C G A C C A T G T C A T A T C A A C A T C A C A T A A A T T A G T C T T G T C T G T T A A T C C G
SARS-CoV    101  A T G A C C A T G T C A T T T C A A C A T C A C A C A A A T T A G T G T T G T C T G T T A A T C C C
MERS-CoV    101  A T G A T C A T G T T A T A G C A A C T C C A C A T A A G A T G G T T T T G T C T G T T T C T C C T
HCoV-229E   101  A T G A T C A T G T G T T T G G C A C T G A T C A T A A G T T C A T T T T A G C T A T T A C A C C A
HCoV-OC43   101  A T G A T C A T G T T A T G G C G A C T G A T C A T A A A T A T G T C T T G A G T G T T T C A C C A
HCoV-NL63   101  A T G A T C A T G T A T T T G G T A C C G A C C A C A A G T T T A T T T T G G C T A T A A C A C C G
HCoV-HKU1   101  A T G A C C A T G T T A T G G C A A C T A A T C A T A A A T A T G T T T T G A G T G T C T C A C C T

SARS-CoV-2  151  T A T G T T T G C A A T G C T C C A G G T T G T G A T G T C A C A G A T G T G A C T C A A C T T T A
SARS-CoV    151  T A T G T T T G C A A T G C C C C A G G T T G T G A T G T C A C T G A T G T G A C A C A A C T G T A
MERS-CoV    151  T A C G T T T G T A A T G C C C C T G G T T G T G G C G T T T C A G A C G T T A C T A A G C T A T A
HCoV-229E   151  T A T G T G T G T A A C A C A T C T G G C T G C A A T G T A A A T G A C G T T A C A A A A C T G T A
HCoV-OC43   151  T A T G T G T G T A A T G C A C C A G G A T G T G A T G T A A A T G A T G T T A C C A A A T T G T A
HCoV-NL63   151  T A T G T A T G T A A T G C A T C A G G T T G T G G T G T T A G T G A T G T C A A A A A A T T G T A
HCoV-HKU1   151  T A C G T T T G T A A T G C A C C T A A C T G T G A T G T G A G T G A T G T C A C C A A A T T A T A

SARS-CoV-2  201  C T T A G G A G G T A T G A G C T A T T A T T G T A A A T C A C A T A A A C C A C C C A T T A G T T
SARS-CoV    201  T C T A G G A G G T A T G A G C T A T T A T T G C A A G T C A C A T A A G C C T C C C A T T A G T T
MERS-CoV    201  T T T A G G T G G T A T G A G C T A C T T T T G T G T A G A T C A T A G A C C T G T G T G T A G T T
HCoV-229E   201  T C T T G G A G G T T T G A A T T A T T A C T G T G T A G A C C A C A A A C C A C A T C T T T C A T
HCoV-OC43   201  T C T A G G T G G T A T G T C A T A T T A T T G T G A A G A C C A T A A G C C A C A A T A T T C A T
HCoV-NL63   201  T C T T G G T G G T T T G A A T T A C T A T T G T A C A A A T C A T A A A C C A C A G T T G T C T T
HCoV-HKU1   201  T T T G G G C G G T A T G T C T T A C T A T T G T G A A A A C C A T A A A C C C C A T T A T T C A T

SARS-CoV-2  251  T T C C A T T G T G T G C T A A T G G A C A A G T T T T T G G T T T A T A T A A A A A T A C A T G T
SARS-CoV    251  T T C C A T T A T G T G C T A A T G G T C A G G T T T T T G G T T T A T A C A A A A A C A C A T G T
MERS-CoV    251  T T C C A C T T T G C G C T A A T G G T C T T G T A T T C G G C T T A T A C A A G A A T A T G T G C
HCoV-229E   251  T C C C A C T G T G T T C A G C T G G T A A T G T C T T T G G T T T G T A C A A A A G T T C T G C T
HCoV-OC43   251  T C A A G T T G G T A A T G A A T G G T C T G G T T T T T G G T C T A T A T A A A C A A T C T T G T
HCoV-NL63   251  T T C C A T T A T G T T C A G C T G G T A A T A T A T T T G G T T T A T A T A A A A A T T C A G C A
HCoV-HKU1   251  T T A A G T T A G T T A T G A A T G G T A T G G T C T T T G G T T T G T A T A A A C A A T C T T G C
```

Figure S3. Sensor S1's target RNA and its upstream and downstream flanking sequences in the *orf1ab* gene of SARS-CoV-2. Homology regions to this sequence from other coronaviruses including SARS-CoV, MERS-CoV, HCoV-229E, HCoV-OC43, HCoV-NL63, and HCoV-HKU1. The red box indicates the homologous sequences of the target region of sensor S1. The blue box denotes the homologous sequences of its upstream and downstream flanking sequences that were synthesized for the cross-reactivity test.

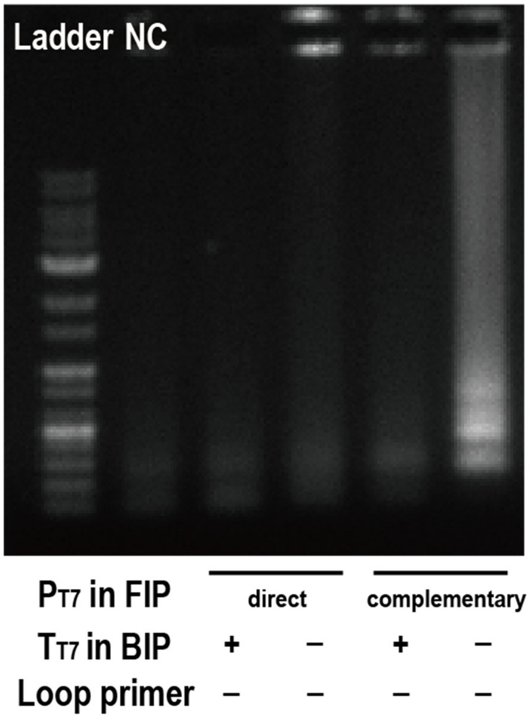

Fig S4. Gel electrophoresis of the modified RT-LAMP products. The ladder-like gel bands are the typical amplification pattern of LAMP. T7 promoter (P_{T7}) was inserted in the FIP primer with a direct or complementary sequence. In the presence of T7 terminator (T_{T7}) in the BIP primer, the RT-LAMP exhibited less amplification. Of note, we used RT-LAMP primers of sensor M6.

Reference

1. Pardee, K.; Green, A. A.; Takahashi, M. K.; Braff, D.; Lambert, G.; Lee, J. W.; Ferrante, T.; Ma, D.; Donghia, N.; Fan, M.; Daringer, N. M.; Bosch, I.; Dudley, D. M.; O'Connor, D. H.; Gehrke, L.; Collins, J. J. Rapid, Low-Cost Detection of Zika Virus Using Programmable Biomolecular Components. Cell 2016, 165, 1255–1266.
